# Sensor organic light-emitting diode display, combining fingerprint and biomarker capturing

**DOI:** 10.1038/s44172-024-00239-8

**Published:** 2024-07-04

**Authors:** Chul Kim, Kwang Soo Bae, Gunhee Kim, Dae-Young Lee, Gyeongub Moon, Dongwook Yang, Hyeonjun Lee, Jongyeop An, Jungwoo Park, Seokgyu Yoon, Cheol Gon Lee, Mu Kyung Jeon, Sanghwan Cho, Sunghan Kim, Yongjo Kim, Changhee Lee

**Affiliations:** Display Research Center, Samsung Display, Giheung, Gyeonggi Republic of Korea

**Keywords:** Electrical and electronic engineering, Technology

## Abstract

Display has been evolving its role as a conventional optical output device into an user interactive input and output device by harnessing various sensors and taking full advantage of its user interaction friendly nature. To demonstrate this phenomenon, here we report a full organic photodiode embedded organic light-emitting diode display as multiple objects sensing platform which identifies the user’s physiological data based on the obtained photoplethysmography signal and also detects a fingerprint for an authentication concurrently in a single device. This paper introduces the technical breakthroughs to solve the complex technical challenges due to the crosstalks induced within the shared common layers during the full integration of the two conflicting devices and also the method made possible for the multiple objects sensing with the measurement results. Consequently, we believe it could prove a progression of display to a fully bidirectional innovative smart user interactive device and also could take a role as a sophisticated future display beyond organic light-emitting diode display.

## Abstract (web summary)

Chul Kim and colleagues show a sensor display which is able to capture biometric data. In one device, they combine fingerprint identification and biomarker estimation while maintaining the same display quality.

## Introduction

Since the first commercialization of Cathode Ray Tube (CRT) in 1922 to the most recently organic light-emitting diodes (OLEDs), the electrically operated display devices have been restrictively around us as a unidirectional optical output device. However, its role has been progressively changing to the bidirectional user interactive device with a proliferation of smart mobile devices, where the intuitive user interaction is mandated. But, its roles still remain to the essential user interactive applications such as a touch, pen, and user authentication etc. with generally laminating an additional stand-alone sensors to the display^1,2^ As a result, it causes a plenty of redundancies in the systems that accompanies increased cost, thickness, and system complexity. Most importantly, it hinders the further evolution of the smart devices.

Display can be the most effective user interactive medium in the electronic systems, as it provides instantaneous impactful visual feedback to the users. Considering its user interaction friendly nature, it will create a tremendous synergy when the display is evolved as the fully bidirectional smart user interactive device with a sophisticated sensing capabilities by embedding heterogeneous sensors. As a result, it can avoid aforementioned redundancies thus provides abundant benefits to the end systems, and also can lead the new evolution of the smart devices. The sensing capabilities can include but not limited to the bio signals, fingerprint, RF signals, user gesture, surrounding objects etc.

Recently, the concept of multi-functional sensors integrated display called the “All-in-one sensor OLED” has been presented^3^. To demonstrate the concept, we implemented the full OPD embedded in the same plane with the OLED, as a multiple objects sensing platform with the capability of identifying user’s physiological data such as a blood pressure (BP), heart rate (HR), heart rate variability (HRV), and cardiovascular health (CH) etc. based on the obtained PPG signal and as well as a fingerprint. Whereas the current state-of-the-art display based sensors only support single object detection (either fingerprint or PPG) with a stand-alone^4–7^ or display integrated^8^ sensor structure. The previous literature^8^, demonstrated the display integrated fingerprint sensor at the prototype level that was separately formed the common layers for the OLED and the OPD by the side-by-side deposition to avoid the crosstalk within the shared common layers, thus faced complicated fabrication steps which is impractical. Sharing the layers between the devices is an extremely complex technical challenge even in the OLED process itself^9^ and it is even worse in the Sensor OLED where the two conflicting devices are integrated. However, it is a mandatory prerequisite to achieve a high level of technical perfection with a superb performance by removing the burdens from the separate deposition thus enables reduced fabrication steps and lead time and also allows high-resolution display implementation. In this paper, we proposed the solutions; the Seperator structure and the sensing method called Alternative Sensing Process (ASP) thus achieved the target performances and secured the completeness of technology that can be mass-produced. In terms of the multiple objects sensing function that is merging the two contradicting technologies (PPG and fingerprint) into a single device was achieved by setting the device structure optimized for fingerprint sensing and compensate the drawbacks for PPG sensing (mainly reduced signal) by the proposed sensing method called Multiple Lines Integration (MLI). In consequence, the Sensor OLED display as a multi-functional optical sensing platform was implemented to be able to suggest a new direction of the display progression thus enables a further evolution of the smart devices.

In this paper, we introduce (1) the concept of the All-in-one Sensor OLED, (2) integration of the OPD and the OLED, (3) technical challenges during the full integration and its solutions to circumvent the issues to enable the technological breakthrough, and (4) the distinctive approaches to achieve the multiple objects sensing capability in a single display device, and 5) the implementation results including the applications aspect of the device such as a fingerprint and a bio sensor. Specifically focused on an underlying sensing principle and its preliminarily validation results as a blood pressure sensor (BPS) with the commercial BP monitor by a pilot trial.

## Results

### Concept of the All-in-one Sensor OLED

Conventionally, the display as an optical output device, its development has been predominantly focused on a high resolution, luminance and refresh rate with a lower power consumption. However, since the emergence of the smart mobile devices, the paradigm has been slightly shifted to the user interactive display, supporting basic user interface functions as mentioned previously. Moreover, the display devices, especially OLED has been further expanding its territories to the more advanced applications including biophotonics, optogenetics^10^, communication field^11^ and beyond, due to the self-emissive characteristic thus enables thinner display with a simpler structure unlike LCD and manufacturing versatility of the substrate from the use of amorphous materials. Supplementary Fig. 1 shows the concept of the All-in-one Sensor OLED, it integrates various heterogeneous sensors and performs the sophisticated sensing operations without scarifying an optical output performance. As an initial step, we fully combined the OPD with the current state-of-the-art flexible OLED display in the same plane while maintaining the same display performance. When the user input their finger on the display, it can optically sense multiple objects including a PPG signal and fingerprint concurrently. Whereas the previous literatures can sense a fingerprint image only with the lower resolution^8^ or PPG signal only from the single pair of separate OLED and OPD^5,6^. Based on the obtained PPG signal, we have expanded further its applications to the more advanced sensing areas such as the multiple biomarkers as will be explained in detail later.

### Implementation of the multi-functional Sensor OLED

**Fig. 1:**
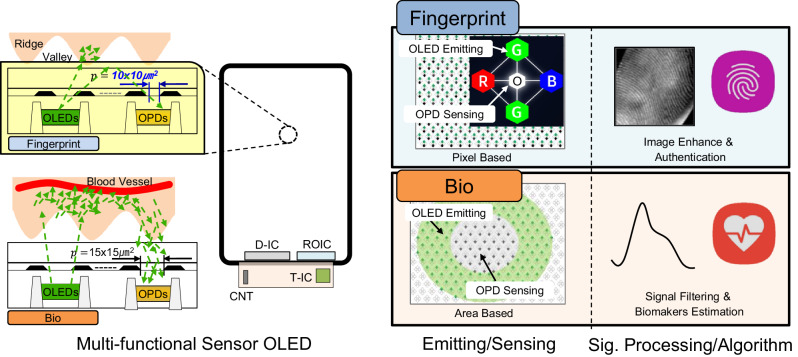
The concept of the multi-functional Sensor OLED. The operation principle of the Sensor OLED is shown. It shares the identical hardware including the Sensor OLED and electronics, but differentiated the emitting, sensing, signal processing, and algorithm depending on the applications. OLED organic light-emitting diode, OPD organic photodiode, D-IC driver-integrated circuit, ROIC read-out integrated circuit, T-IC touch integrated circuit, CNT connector.

Figure 1 shows the concept of the multi-functional sensor OLED, it has an identical hardware including the Sensor OLED and the electronics to support its operation but differentiate the emitting, sensing, signal processing and algorithm depending on the applications. The details will be explained later.

In terms of the Sensor OLED, as shown in the figure, the integration of the OPD with the OLED in the same horizontal plane was carried out. This section introduces two main implementation aspects among others that are (1) a light selection for an optical sensing and (2) the OPD integration into the OLED^12^.

Firstly, in terms of the light selection as a light source perspective, green has the highest emission efficiency in the display, which is advantageous to acquire a high signal-to-noise ratio (SNR) signal. As a sensing perspective, it has a high skin absorption coefficient^13^ thus it is beneficial to obtain a better contrast signal both in fingerprint and PPG. In terms of PPG application, it also has a strong signal immunity to temperature change as of the shorter penetration depth^14^. Most importantly, as shown in the Supplementary Figs. 3 and 4, it achieves high amplitude signal at a lower contact force of less than 11kPa as of the external pressure effect^15^ thus achieves best SNR signal with the user’s gentle contact to the sensor. However, red light enforces to the user to maintain a constant contact force (~14kPa) to guarantee a high quality signal which is disadvantageous to the real user scenario. Moreover, the adoption of the green light in the commercial markets are prevalent, especially in the PPG applications. Therefore, the green light had chosen as a target light in the manufactured sensor, despite of its weakness such as a skin tone effect (dark skin absorbs more green light).

**Fig. 2:**
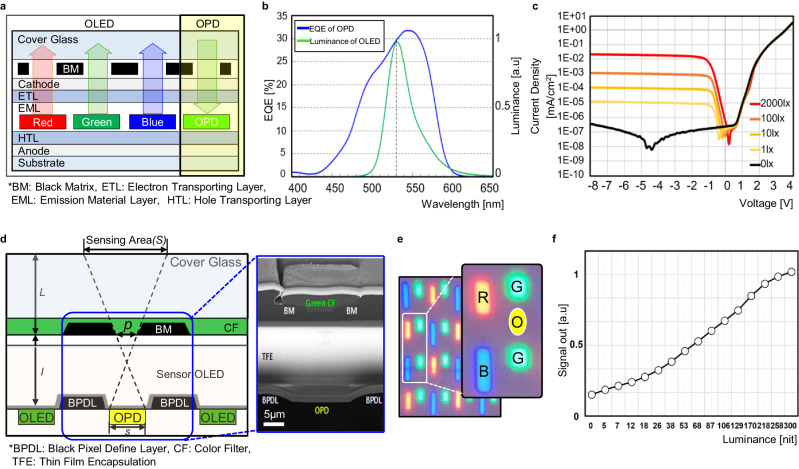
The implemented multi-functional Sensor OLED. **a** The cross-sectional view of the Sensor OLED. **b** Overlapped emission spectrum of the OLED and absorption spectrum (EQE) of the OPD. **c** OPD I-V characteristic curve depending on the changed illuminance (0–2000 lx). **d** The vertical structure of the Sensor OLED along with the optical system and image of the implemented Sensor OLED. **e** Top view image of the implemented Sensor OLED pixels.(Supplementary Fig. 2) **f** OLED light intensity vs OPD current output from the implemented Sensor OLED. OLED organic light-emitting diode, OPD organic photodiode.

The OLED green light has an emission peak at a wavelength of 530 nm and it was selected as a light source for the sensing operation thus removes an additional need for the external light sources which is obliged in the conventional off-the-shelf optical sensors. It could be an additional benefit from the display and sensor integration. Therefore, it becomes crucially important to maximize the spectral overlap between the OLED emission and the OPD absorption, so as to obtain the best achievable sensing performance. Figure 2b shows the normalized spectral characteristics of the emission spectrum of the OLED and the absorption spectrum (External Quantum Efficiency (EQE)) of the OPD. As can be seen in the figure, the absorption spectrum of the OPD covers broadly the emission spectrum of the OLED thus allows to maximize the sensing efficiency. Additionally, due to the wavelength selectivity of the OPD (sensible range between 400 ~ 600 nm, because of the combined effects from the OPD materials and the color filter), the additional Infrared Radiation (IR)-cut filter which is mandated in the conventional optical sensors can be removed.

Secondly, the OPD integration into the OLED will be explained. The Sensor OLED was manufactured with the identical OLED processes and materials, except the custom-designed OPD. The OPDs are allocated in-between the display pixels in the entire display active area. So it is inevitable that the OPD shares the common layers (Electron Transporting Layer (ETL), Hole Transporting Layer (HTL)) with the OLED as shown in the Fig. 2a which shows a cross-sectional view of the Sensor OLED. And also the light-absorbing layer in the OPD was implemented in the same plane with the OLED Emission Material Layer (EML). After the organic layers are successfully evaporated, encapsulation process is followed using the thin film called Thin Film Encapsulation (TFE). The touch layers are formed on top of the TFE. Black Matrix (BM) and Color Filter (CF) array processes are followed by the succeeding module processing including a bonding the display electronics and laminating the cover glass. The OPD’s characteristic of photocurrent vs voltage curve depending on the changed illuminance is shown in the Fig. 2c. It shows a low leakage current and a linear photocurrent with the increased illuminance which is suitable for the multi-functional Sensor OLED.

Figure 2d illustrates a vertical structure of the Sensor OLED. In the figure, the concept of the optical system structure is described. As the OLEDs and OPDs are formed at the same plane, the BM for the display was re-used as the optical system for the OPD which controls the amount of sensing area and light input. The optical sensing area *(S)* of the Sensor OLED is solely determined by the factors which encompasses the thickness of the top stack-up (*L*) and the bottom stack-up (*l*), the opening diameter of the BM hole (*p*), and the OPD width (*s*)^8^. To support the multiple object sensing, the dimensions of each factor have to be carefully decided, which will be explained later. Figure 2d also shows the vertical structure of the implemented Sensor OLED. The photo of the implemented Sensor OLED’s pixels is shown in the Fig. 2e. As shown in the figure, the OPD pixels are located in-between the RGBG display pixels in the same horizontal plane. Figure 2f shows the OPD output linearity according to the varying OLED light intensity which shows a linear trend and suitable for the multiple objects sensing. The details of the OPD can be founded in refs. ^8,16,17^.

### Technical challenges

Since the two contradicting devices (OLED, OPD) are integrated into the single display device thus shares the identical layers, it is inevitable to face crucial technical challenges. In this section, the two predominant contributors to degrade the overall performance and the countermeasures being applied will be explained.

**Fig. 3:**
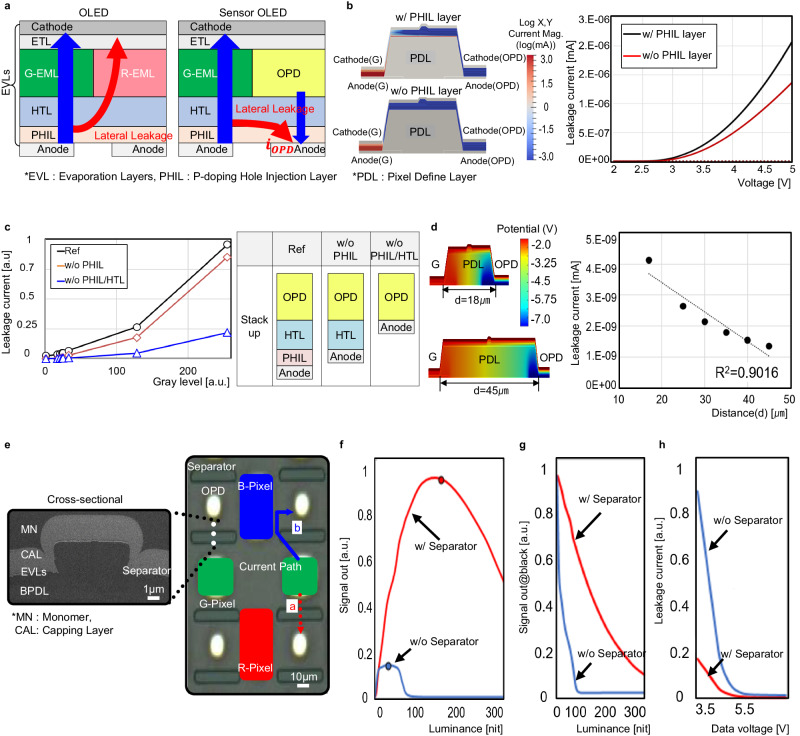
The technical challenges: (1) electrical leakage. **a** The concept of the induced electrical leakage current. **b** Simulation result to identify the effect of the common layers. **c** Measurement results from the manufactured Sensor OLED with the different layer structures. **d** Simulation result to identify the effect of the leakage depending on the distance. **e** The implemented Separator structure. **f**–**h** The measurement results of the Separator. OLED organic light-emitting diode, OPD organic photodiode, EML emission material layer, HTL hole transporting layer, BPDL black pixel define layer.

Firstly, the electrical leakage as illustrated in the Fig. 3a will be introduced. The OLED is operate in the forward bias condition, whereas the OPD is operate in the reverse bias condition. As a result, due to the voltage difference between the OLED and OPD’s anode, it induces the parasitic current as a crosstalk, through the shared common layers (HTL, P Hole Injection Layer (PHIL)) as shown in the Fig. 3a. To validate the exact cause of the parasitic current, the simulations and measurements were executed. The Sensor OLED was modeled and simulated by the custom-developed analysis tool. As shown in the Fig. 3b, the common PHIL layer contributes the parasitic leakage current. It was also verified with the measurement results from the fabricated Sensor OLEDs with the different layer configurations. As shown in the Fig. 3c, the common layers provide a vast amount of the leakage currents thus affects the photo current generated by the OPD. The straightforward solution to alleviate the current is physically disconnect the common layers and increase the distance between the OLED and OPD which was applied in ref. ^8^ with the drawbacks such as complicated fabrication steps, increased lead time and prevent high-resolution implementation. In Fig. 3d shows the leakage current simulation results depending on the OLED and OPD distance. As can be seen from the figure, the longer the distance results in reduced the current. However, the increased distance makes hard for the physical integration of the OLED and OPD within the dedicated space (display active area) without sacrificing the resolution. As a result, we proposed the organic Separator structure with the reversed taper shape as shown in the Fig. 3e. It was formed on top of the Pixel Define Layer (PDL) and surround the OPD to physically disconnect the common deposition layers (HTL, PHIL) between the OLED green pixel and OPD. Thus, the distance between two devices was increased from *a* = 21 μm to *b* = 42 μm as shown in the Fig. 3e which results reduced the current. Figure 3f–h shows the effectiveness of the Separator. Figure 3f shows the sensor signal depending on the varying luminance. The Separator can effectively reduce the leakage, thus obtains a wider luminance dynamic range with larger signal, whereas the without Separator shows a limited dynamic range in the low luminance area with much smaller signal as it is saturated early due to the large leakage current induced. Figure 3g, h also explain the linear signal drop and lower leakage were observed with the Separator structure, on the other hand the sudden signal drop in the low luminance area and larger leakage were witnessed in the without Separator structure. As a result, the lateral leakage was effectively eliminated by adopting the proposed Separator structure thus guarantee a maximum signal sensing performance in the Sensor OLED.

**Fig. 4:**
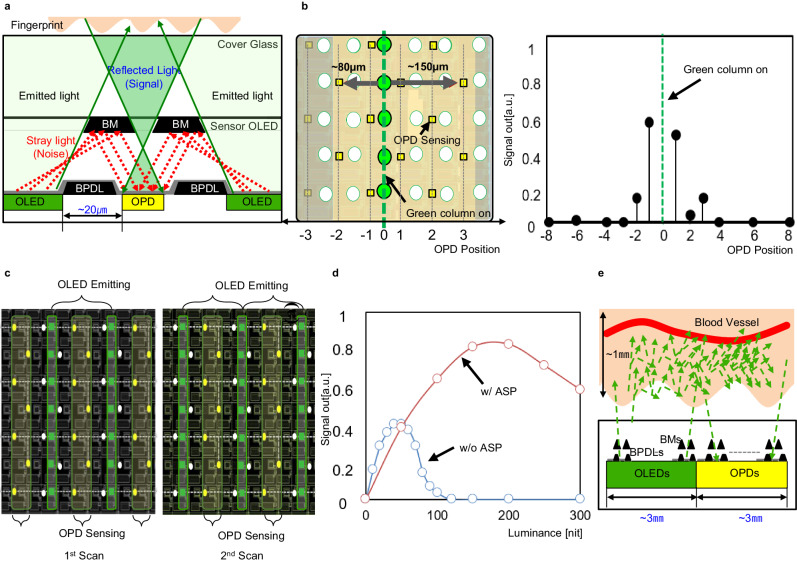
The technical challenges: (2) optical leakage. **a** The concept of the induced optical leakage current. **b** Experiment concept and measurement result to identify the effect of the stray light. **c** Operation concept of the alternative sensing process (ASP). **d** Measurement result of the ASP. **e** Optical leakage immunity during the PPG sensing. OLED organic light-emitting diode, OPD organic photodiode, BM black matrix, BPDL black pixel define layer.

The second challenging issue is a stray light-induced leakage current what we called an optical leakage. As shown in the Fig. 4a, the trapped stray light inside the panel affects a photo signal current in the OPD thus acts as a noise which has to be removed. However, it is very hard to quantitative characterize the amount of the trapped stray light, as of its angular dependency in OLED emission light and OPD EQE, thus leads a certain limitation to control it. To accurately characterize the effect of the stray light, the experiment as shown in the Fig. 4b was carried out. The one single-column line of green OLED pixels was turned on with the black reflector fully covered the Sensor OLED to identify the stray light component only. Then the signals from the adjacent columns of OPD were measured respectively. Table 1: fingerprint vs PPG sensing

**Table 1:** Fingerprint vs PPG sensing

Item		Fingerprint	PPG
Sensing	Object	Fingerprint (Ridge, Valley)	Arterial volume
	Method	Pixel based	Area based
Key factor		High spatial resolution and contrast ratio	High contrast ratio
Frame rate		Still image(2–3 Hz)	Video image (≥30 Hz)
Optical system		Required	Not required
Signal processing		Image processing	Band-pass filtering
Algorithm		Authentication	Biomarker estimation

As shown in the figure, the maximum stray light was observed in the 1st adjacent column then from the 2nd column, the effect was minimized. The observed sensing data asymmetry between left and right in the Fig. 4b right was caused by the asymmetrically formed OPD sensing lines (coupling cap.), which induces a different amount of crosstalk during the display data transition. It can be improved by adopting a shield layer or even sensing line distribution.

To take full advantage of this phenomenon, we proposed the Alternative Sensing Process (ASP) as illustrated in the Fig. 4c. The evenly spaced columns of green OLED pixels were alternatively turned on then the OPDs in the 2nd and 3rd adjacent columns from the turned on OLED columns were sensed concurrently, then it repeated twice so that the full frame of the OPD signal can be obtained. Figure 4d shows its effectiveness, as can be seen the figure, the optical leakage was successfully removed when the ASP was applied. However, it has a disadvantage of twofold increased scan time. The proposed ASP is especially effective where the pixel-based OLED emission and OPD sensing is required such as a fingerprint sensing. The efficiency can be trivial in the area-based sensing applications such as a PPG, as the OLED emission and OPD sensing area are separated as illustrated in the Fig. 4e. In the Sensor OLED, the ASP was restrictively applied depending on the applications. In conclusion, by applying the proposed countermeasures, we secured the performance as well as the completeness of technology.

### Multiple objects sensing OLED

Table 1 shows the differences between fingerprint and PPG sensing. The sensing objects, method, key performance measures, frame rate and the need for the optical system are the main differences which is somewhat contradicting each other. Figure 1 shows the sensing principles. Fingerprint sensing is done by detecting the reflected light difference between the fingerprint ridge (diffuse reflection) and valley (specular reflection) from the emitted OLED light. While the PPG sensing is done by detecting the volumetric changes in the blood vessel from the regular heart palpitations by discriminating the intensity of the scattered light absorbed. To enable the multiple objects sensing, we made technological innovations throughout the entire constituent blocks in the system; from the display device, the electronics to operate the Sensor OLED, including the display driver-integrated circuit (IC) and the optical readout IC (ROIC), to the dedicated signal processing techniques and algorithms.

**Fig. 5:**
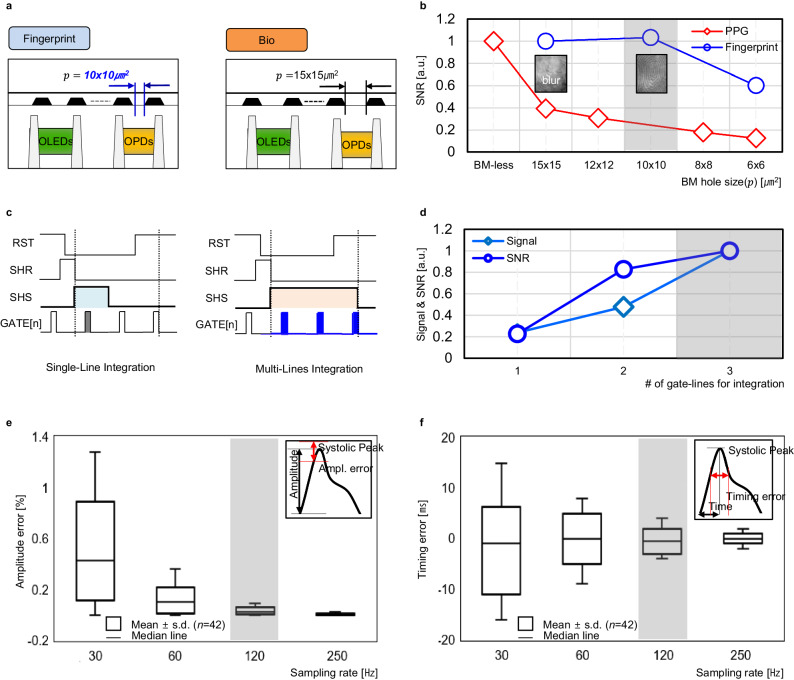
The multiple objects sensing OLED. **a** The optical systems for fingerprint and PPG. **b** The effect of the optical system: BM hole size vs SNR (Fingerprint, PPG). **c** Signal sensing methods (single-line and multi-lines integration). **d** Effectiveness of the MLI. **e**, **f** PPG sampling rate vs amplitude error and timing error obtained from the implemented PPG sensor with the stand-alone off-the-shelf components. (Mean was obtained by adding up the values and divided by the number of data (*n* = 42). Standard deviation was derived from the square root of the variance. (Sum of the squares of the differences from the mean divided by the total number of data minus one) Median is the middle value in a data set, The detailed methods and results can be found in the Supplementary Fig. 5). OLED organic light-emitting diode, OPD organic photodiode.

The entire system block diagram is shown in the Fig. 1. To manufacture the Sensor OLED, first of all, the OPD size has to be carefully decided between its area and sensitivity. Once it is fixed then the diameter of the BM hole (*p*) as the optical system is need to be selected to support the multiple objects sensing as shown in the Fig. 5a. It has to have an optimum size to identify the small different between fingerprint’s ridge (R) and valley (V) signals that is $10\times 10$μm^2^ in the implemented sensor as the larger or smaller than that causes an image blurring (from the increased sensing area*(S)* thus affects signal interference between the R and V) or SNR degradation (from the blocking the input light). However, it limits the amount of lights adversely, when it is used as the PPG sensor as shown in the Fig. 5b. To enable the multiple objects sensing capability, it is inevitable that the BM hole diameter (*p*) was chosen to be the one optimized for the fingerprint sensing($10\times 10$μm^2^) in the entire display active area. The signal degradation during the PPG sensing, was compensated by our proprietary Multiple Lines Integration (MLI) sensing technique in the ROIC that is integrate signals from the OPDs in the several consecutive gate lines into the feedback capacitor at the charge amplifier in the ROIC to increase SNR, as the spatial resolution is not a major concern in the PPG sensing. In contrast, the signal from the each horizontal single gate line connected OPD is integrated in the corresponding single integration window at the ROIC, as the high spatial resolution of the image is a main performance measure in the fingerprint application. The sensing principle and operation timing of the MLI is shown in the Fig. 5c and its effectiveness is also shown in the Fig. 5d, as the SNR is increased in proportion to the number of signal integrations. Afterward, the preprocessing for the image enhancement is execute then the manipulated signal goes into the authentication algorithm to identify the users in the fingerprint sensing application. Whereas, the signal undergo a low pass filtering then become an input of the multi-biomarkers estimation algorithm to sense the biomarkers in the PPG sensing application. The OLED emitting and the OPD sensing areas and also the ROIC sensing timing are determined depending on the applications. Figure 1 also shows the OLED and OPD sensing pattern during the PPG and fingerprint sensing. The circle shape with the diameter of 9 mm achieved the best SNR in the PPG sensing. While the full individual OLEDs and OPDs are emitting and sensing in the fingerprint application. In the PPG sensing, determine the sampling rate is another important design choice, as the lower sampling rate means more quantization error in the PPG signal thus affects the accuracy of the bio sensing. Figure 5e, f show the systolic peak errors in the PPG pulse trains depending on the varying sampling rates of the pulse. As can be seen in the figure, the amplitude and timing error are worsening when the sampling rate is less than 120 Hz which requires an additional signal reconstruction such as an interpolation. So, the sampling rate of 120 Hz was chosen during the PPG sensing, as the OPD in the Sensor OLED share the identical scan circuits with the display which also operates with the refresh rate of 120 Hz. Moreover, it also eliminates the need for the additional signal processing as previously stated. As a result, we had successfully achieved the multiple-objects sensing capability.

The mock-up system with the fabricated Sensor OLED was implemented as shown in the Supplementary Fig. 6a–d. Supplementary Figure 6b shows its operation examples of the fingerprint sensing application which can sense multiple fingerprints scanning and also image scanning in the entire display area. In the Supplementary Fig. 6c, the operation example of the multi bio-markers sensing is shown, currently its function is limited to sense a BP, HR, HRV, and CH only, but it will be expanded further in the future such as a respiratory rate, blood vessel elasticity, oxygen saturation, and more. The mock-up system architecture is shown in the Supplementary Fig. 6d.

### Implementation results

**Fig. 6:**
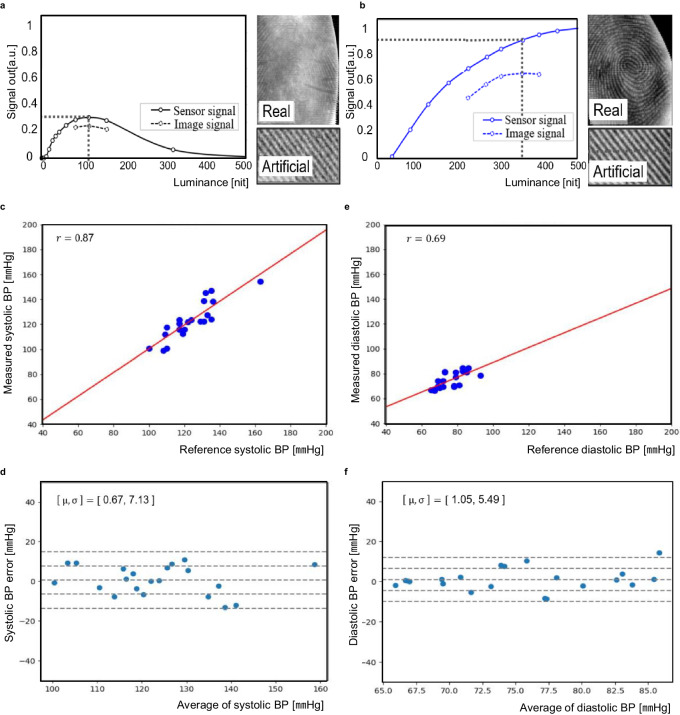
The implementation results. **a** Fingerprint SNR without the applied countermeasures (the Separator and the ASP). **b** Fingerprint SNR with the applied countermeasures. *Sensor signal is the code difference between black and white images obtained while the reflectors are on the Sensor OLED. It represents the full dynamic range of the OPD. Image signal is the code difference between ridge and valley images obtained while the artificial finger is placed on the Sensor OLED. It indicates the signal range of the OPD in the fingerprint application (Supplementary Fig. 7). **c**–**f** The pilot study results for the BP sensor validation: correlation and Bland-Altman plots comparing the reference BP with the Sensor OLED measurement.

As shown in the Supplementary Fig. 6, the multiple objects sensing OLED has been successfully implemented and demonstrated its operation and also the video in the Supplementary Movie 1 demonstrates the results as well. Figure 6a, b show the fingerprint sensing results from the implemented Sensor OLED without and with the applied countermeasures respectively. The concept of the image and sensor signal are employed to show the OPD’s full dynamic range and the signal range in the fingerprint application respectively. The methodology being adopted is described in the Supplementary Fig. 7 and fingerprint sensing section in the Methods. Higher SNR and wider luminance dynamic range was achieved as a result of the applied proposed countermeasures. The maximum SNR of 8.5 was achieved at 350nit, thus obtained ≤1% of false rejection rate (FRR) when false acceptance rate (FAR) value is 1/100,000 which satisfies the mandated security level. Moreover, the larger the sensor size can enhance the security level and also the multiple fingers up to 10 concurrent authentication can lead unbreakable security level as shown in the Supplementary Fig. 8.

In terms of bio-sensing applications, the details about the BPS will be explained including a brief introduction of the sensing principle and preliminary validation results. Supplementary Fig. 9 illustrates the sensing principle of the multi-biomarkers. Based on the PPG pulse trains obtained, the distinct pulse features, period, peak-to-peak variations, low-frequency information etc. are extracted to estimate the relevant biomarkers.

As hypertension is a silent killer and prevalent worldwide, its management by the ubiquitous BP monitoring is crucially important to the people with the related symptoms. If the smartphones, which is an indispensable device to life, support BP monitoring, then it can provide a tremendous benefit to maintain BP by allowing the user to ubiquitously monitoring the BP. Thus, it became our motivation to develop the Sensor OLED with the BP monitoring capability. Supplementary Figure 9 also explains the underlying BP sensing principle. The BP monitoring technologies can be categorized depending on the necessity for calibration. The pulse wave analysis (PWA), facial video processing (FVP), and volume control (VC) are the main technologies adopted in the market. However, the PWA^18,19^ which is meticulously optimized for the PPG waves obtained from the Sensor OLED with the help of the machine learning (ML) was employed as it is more suitable for interfacing with the Sensor OLED (since, FVP and VC require an additional smartphone camera and cuff). The brief introduction about the BP estimation technologies is summarized in the Supplementary Table 1. To check its initial feasibility as a BPS, the pilot trial was executed with the total of 32 participated users. (age: 38.8 ± 11.3 years, height: 169 ± 7.7cm, weight: 63 ± 12.2kg, Male: 51%, Female: 49%). The data from 26 users and 6 users were used for a learning set and a test set for the machine learning algorithm respectively. Supplementary Figure 10 depicts the trial environment, the commercial cuff oscillometry BP monitor (Omron HEM-7121) and the implemented system with the Sensor OLED were used as a reference and a test device respectively. The measurements were executed simultaneously as shown in the figure. Figure 6c–f shows the study results, the accuracy of the Sensor OLED-based system obtained the mean and standard deviation errors of 0.67 and 7.13 mmHg for a systolic BP and 1.05 and 5.49 mmHg for a diastolic BP which is within the range of the accuracy requirements for the commercial BP monitor that are ≤±5 and 8 mmHg. As a result, it clearly demonstrates the Sensor OLED’s feasibility as a BPS. The formal clinical evaluation is scheduled to carry out in the near future by strictly following the standardized protocols^20,21^ to fully validate its performance as a BPS. Table 2 tabulates the performance comparison with the current state-of-the-art OPD-based sensors. As shown in the table, the implemented Sensor OLED demonstrates superior performance in its size, resolution, supportable functions, and technological level of completion.

**Table 2:** Performance comparison

Item		[5]	[6]	[7]	[8]	This work
Type		Standalone sensor (OLED/OPD, Patch)	Standalone sensor (OLED/OPD, Patch)	Standalone sensor (OPD only, Patch)	Sensor OLED (OLED/OPD, Panel)	Sensor OLED (OLED/OPD, Panel)
Size		–	–	1.57”	3.07”	7.5”
Resolution	Display	17x7	–	–	360x540 (212ppi)	2160 × 1780 (374ppi)
	Sensor	Single Channel PPG	Single Channel PPG	508ppi	212ppi	262ppi
Function		PPG (HR)	PPG (SpO_2_)	Fingerprint	Fingerprint	PPG(BP,HR,HRV,CH), Fingerprint

Lastly, the polarizer on top of the Sensor OLED causes a signal degradation in both fingerprint and PPG sensing. In case of fingerprint sensing, the degradation appears differently between the ridge and valley, however, the signal (the difference between the ridge and valley) is still comparable despite of the degradation. During PPG sensing, it was compensated by adopting the MLI sensing technique as previously stated.

## Conclusions

We presented the Sensor OLED, which has been successfully evaporated the OPD within the OLED pixels, without further vertical expansion of the panel. Thus, the benefits such as the full area sensing capability within the display active region and the slimmest display module were achieved. Based on the implemented Sensor OLED, we also have successfully demonstrated the display that identifies the user’s physiological data and also a fingerprint. As a result, we strongly believe, the presented multi-functional Sensor OLED display could demonstrate the future display progression and also could accelerate a further evolution of the smart devices.

## Methods

### PPG amplitude vs contact pressure

The system made by the off-the-shelf components (LED (Osram, SFH7013), PD (Vishay, VEDM8080), Readout IC (Maxim, MAX86171)) and the scale (CAS, WZ-3A) was used for the experiment to decide the target wavelength. Linearly incremented contact pressure was applied from the finger with a different wavelength of the light and the corresponding PPG out pulses were measured.

### Implementation of the Sensor OLED

It was manufactured with the identical OLED processes and materials, except the custom-designed OPD. It was implemented based on the state-of-the-art flexible display (size: 7.5″, display resolution: 2160 × 1780). On top of the polyimide (PI) substrate, oxide and low-temperature polycrystalline silicon (LTPS) hybrid Thin-film transistor (TFT) backplane process was done with the conventional OLED processes including deposition, photolithography, etching, and stripping etc. The OPD pixels were designed to allocate in-between the display pixels thus allows the massive array configuration of the OPD and OLED within the display active area. It also shares the common layers with the OLED such as the HTL, ETL. The only difference is the EML in the OLED is replaced by the absorption layer in the OPD. After the organic layers were successfully evaporated, encapsulation process was followed using the thin film called thin film encapsulation (TFE). The touch layers were formed on top of the TFE. After that, organic material for BM was coated then patterned by the photolithography and the identical processes for CF were repeated (coating R/G/B CF organic materials in the corresponding pixel area then patterned by the photolithography). The green CF was formed on top of the OPD, so as to maximize the green light absorbance. Once the panel was ready, the additional module processes such as a bonding the display electronics and laminating the cover glass were followed.

### OPD EQE measurement

The OLED emission spectrum was measured by the spectrophotometer (Topcon, SR-3AR) while emitting the OLED green light. The absorption spectrum was measured the OPD output current by the source meter (Keithley 2635B) while the xenon lamp (SimuLight, LP-Xe 300 W) as a light source was turned on.

### OLED light intensity vs OPD current output

The OLED light intensity varied by changing the preset register values in the D-IC, and the corresponding OPD output was measured by the readout IC in the darkroom environment with the black-and-white reflector on the Sensor OLED.

### System development

The implemented system was comprised of the Sensor OLED, Field Programmable Gate Array (FPGA) board, and PC. The FPGA board was used for controlling and communicating data between the Sensor OLED and PC. A Universal Serial Bus (USB) connection was employed between the FPGA board and PC for a data communication. The software was developed by C# and Python. A custom software made by C# was used to control the FPGA, the Sensor OLED, and the fingerprint authentication algorithm. Python programmed algorithm was used for the biomarkers sensing.

### Fingerprint sensing

Sensor signal was measured with the black and white reflectors on the Sensor OLED. The signal is considered the difference between black and white, and the time noise is considered as a noise. It represents the full dynamic range of the OPD. Image signal was obtained from the human finger and the artificial finger. The code difference between ridge and valley is considered as a signal, and time and spatial noise become a noise. The artificial finger with the 400 μm line pitch was used, and the same principle is applied to derive SNR. It indicates the signal range of the OPD in the fingerprint application. Supplementary Figure 7 illustrates the details.

### Blood pressure sensing

To demonstrate a feasibility, the pilot trial was performed which was approved by the research ethics committee at Samsung Display. The experimental procedure was explained, and a written informed consent was obtained from all participants. A total of 32 users (age: 38.8 ± 11.3 years, height: 169 ± 7.7 cm, weight: 63 ± 12.2 kg, male: 51%, female: 49%) have participated. The tests were performed with concurrent measurements of the commercial cuff oscillometry BP monitor (Omron HEM-7121) and the implemented system with the Sensor OLED. A total of 9 consecutive tests were done by each user. The data from 26 users and 6 users were used for a learning set and a test set for the machine learning algorithm respectively. The results were analyzed to access accuracy in terms of correlation with the correlation coefficient (r) and Bland-Altman plots with the mean (μ), and standard deviation error (σ).

### Supplementary information

Supplementary Material

Description of Additional Supplementary Files

Supplementary Movies 1

## Data Availability

The data that support this study are available from the corresponding authors upon reasonable request.
