# Supplementary material for: Sensor organic light-emitting diode display, combining fingerprint and biomarker capturing
Source: Commun Eng. 2024 Jul 4;3:92. doi: 10.1038/s44172-024-00239-8 (PMC11224248; doi:10.1038/s44172-024-00239-8)
Supplement: Supplementary file 1 — Supplementary Material [file 44172_2024_239_MOESM1_ESM.pdf]

Supplementary Materials for

Sensor organic light-emitting diode display, combining fingerprint and biomarker capturing

Chul Kim, Kwang Soo Bae, Gunhee Kim, Dae-Young Lee, Gyeongub Moon, Dongwook Yang, Hyeonjun Lee, Jongyeop An, Jungwoo Park, Seokgyu Yoon, Cheol Gon Lee, Mu Kyung Jeon, Sanghwan Cho, Sunghan Kim, Yongjo Kim and Changhee Lee

Display Research Center, Samsung Display, Giheung, Gyeonggi, Republic of Korea

This file includes:

Supplementary Figures1 to 10

Supplementary Table1

Supplementary References

All-in-one Sensor OLED

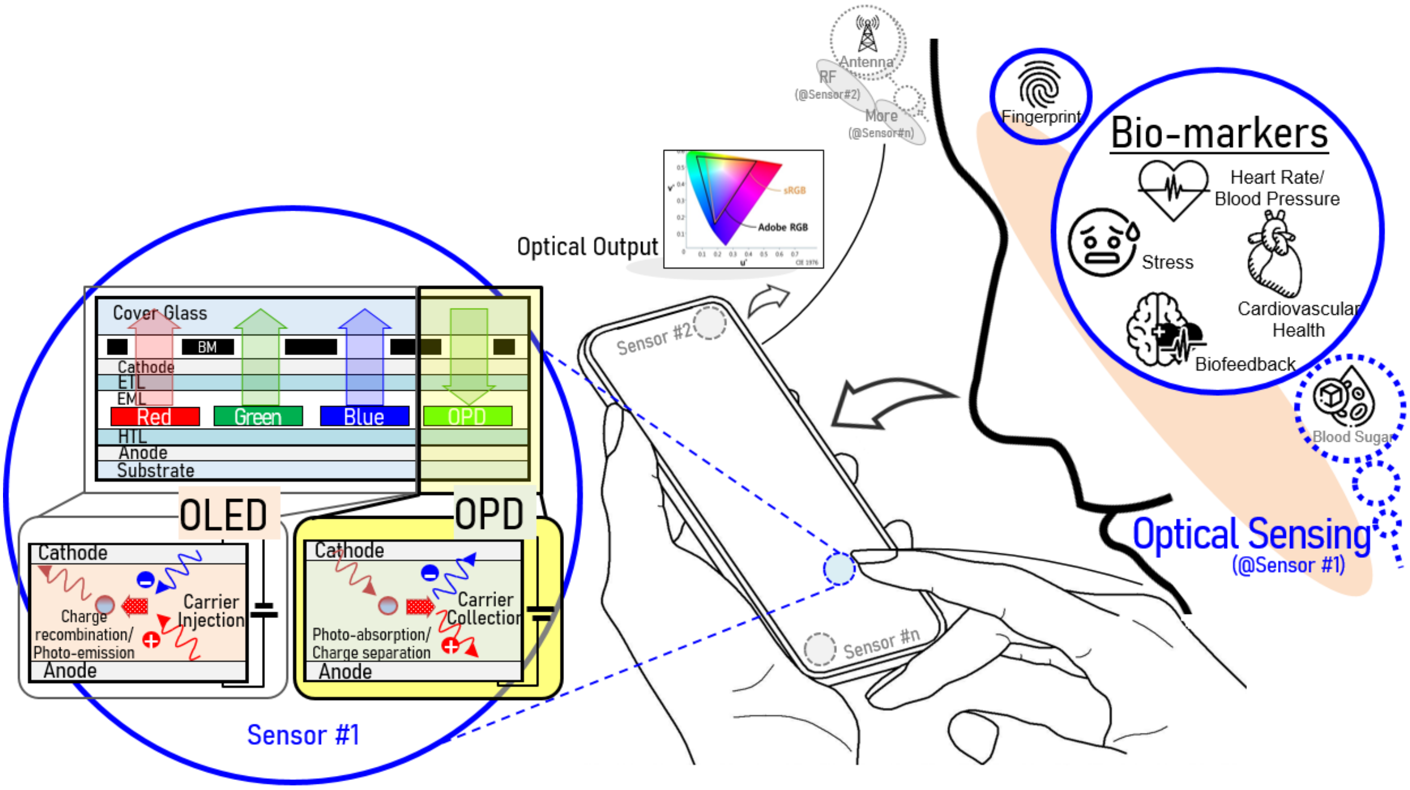

*ETL: Electron Transporting Layer,
EML: Emission Material Layer, HTL: Hole Transporting Layer

Supplementary Figure 1: The concept of the All-in-one Sensor OLED. Display that can sense the user's physiological data, fingerprint, RF signal, user gesture and surrounding objects

Multi-functional Sensor OLED

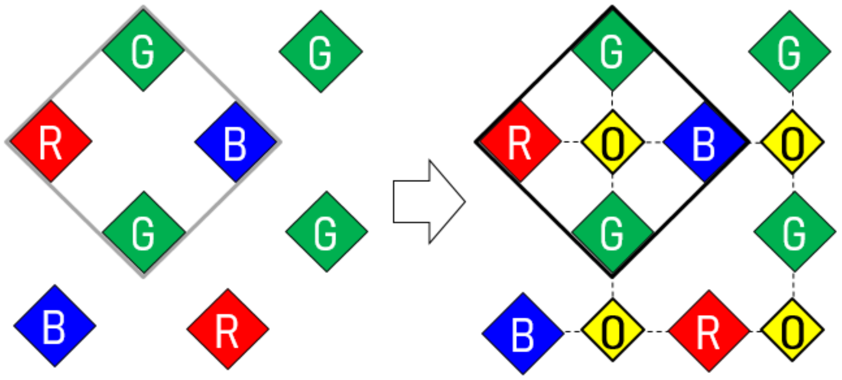

Supplementary Figure 2: The concept of the Multi-functional Sensor OLED. Concept image of the conventional display and the Sensor OLED pixel structure

PPG amplitude Vs Contact Pressure

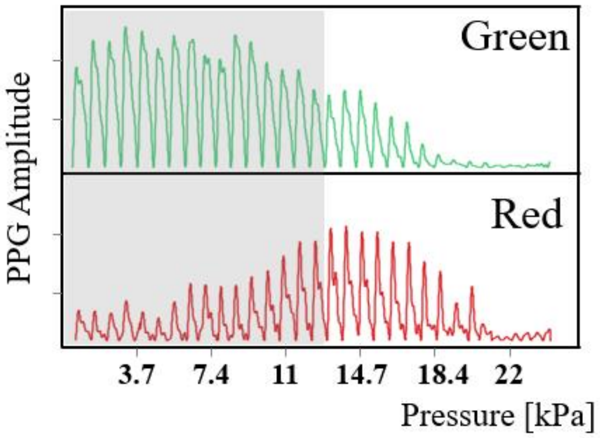

Supplementary Figure 3: PPG amplitude Vs contact pressure depending on the wavelength from the implemented PPG sensor with the stand-alone off-the-shelf components (Supplementary Fig. 4)

Experiments with the stand-alone type PPG Sensor

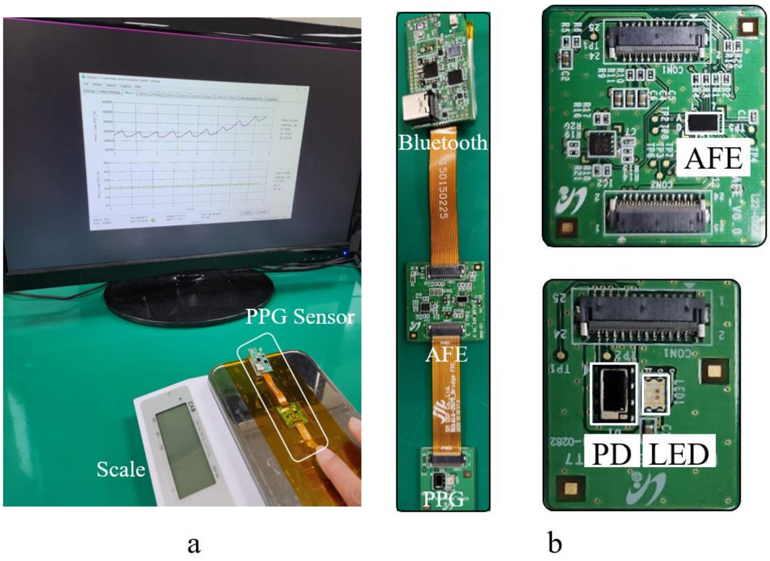

Supplementary Figure 4: The experiment environment for the PPG amplitude vs contact pressure depending on the wavelength.

a, Test environment: The custom made PPG sensor with the standalone off-the-shelf components (LED: Osram SFH7013, PD: Vishay VEMD8080, ROIC Analog front end (AFE): Maxim MAX86171) was used. Linearly incremented contact pressure was applied from the finger with a different wavelength of the light (Green, Red) and the corresponding PPG out pulses were measured. The linear pressure was checked from the scale (CAS, WZ-3A). **b,** The custom made PPG sensor with the LED/PD board and AFE board with the aforementioned off-the-shelf components.

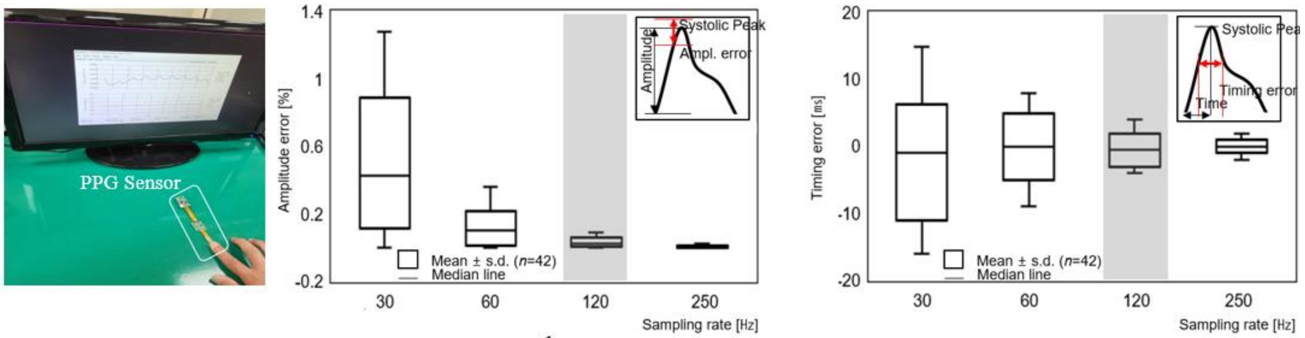

a b c

Supplementary Figure 5: Experiment for the PPG sampling rate Vs amplitude & timing error. a, The PPG signal was sensed during 120s from the finger with the custom made PPG sensor. The signal was obtained with the sampling rate of 1㎑, quantization of 19.5bit from the green light source. The obtained signal was band pass filtered (0.5~3.5㎐) then down sampled to 250, 120, 60, 30㎐ respectively. The amplitude and timing error of the systolic peak were analyzed. **b,** Amplitude error of the systolic peak was calculated from AE= (Amp_{1KHz} - $Amp_{downsampled}$) / Amp_{1KHz}. The amplitude mean error (AME) and amplitude standard deviation (ASD) were AME=0.5%, ASD=0.42% at 30Hz down sampled signal, AME=0.12%, ASD=0.11% at 60Hz down sampled signal, AME=0.03%, ASD=0.03% at 120Hz down sampled signal and AME=0.01%, ASD=0.01% at 250Hz. **c,** Timing error of the systolic peak was calculated from TE=$Time_{1KHz}$ – $Time_{down\ sampled,}$ The time absolute mean error (TME) and time absolute standard deviation (TSD) were TME=8.6ms, TSD=4.6ms at 30Hz down sampled signal, TME=4.2ms, TSD=2.7ms at 60Hz down sampled signal, TME=2.2ms, TSD=1.3ms at 120Hz down sampled signal and TME=0.8ms, TSD=0.7ms at 250Hz down sampled signal. The design trade-off was made between the errors and sampling rate, thus the sampling rate of 120Hz was decided.

Implemented Sensor OLED

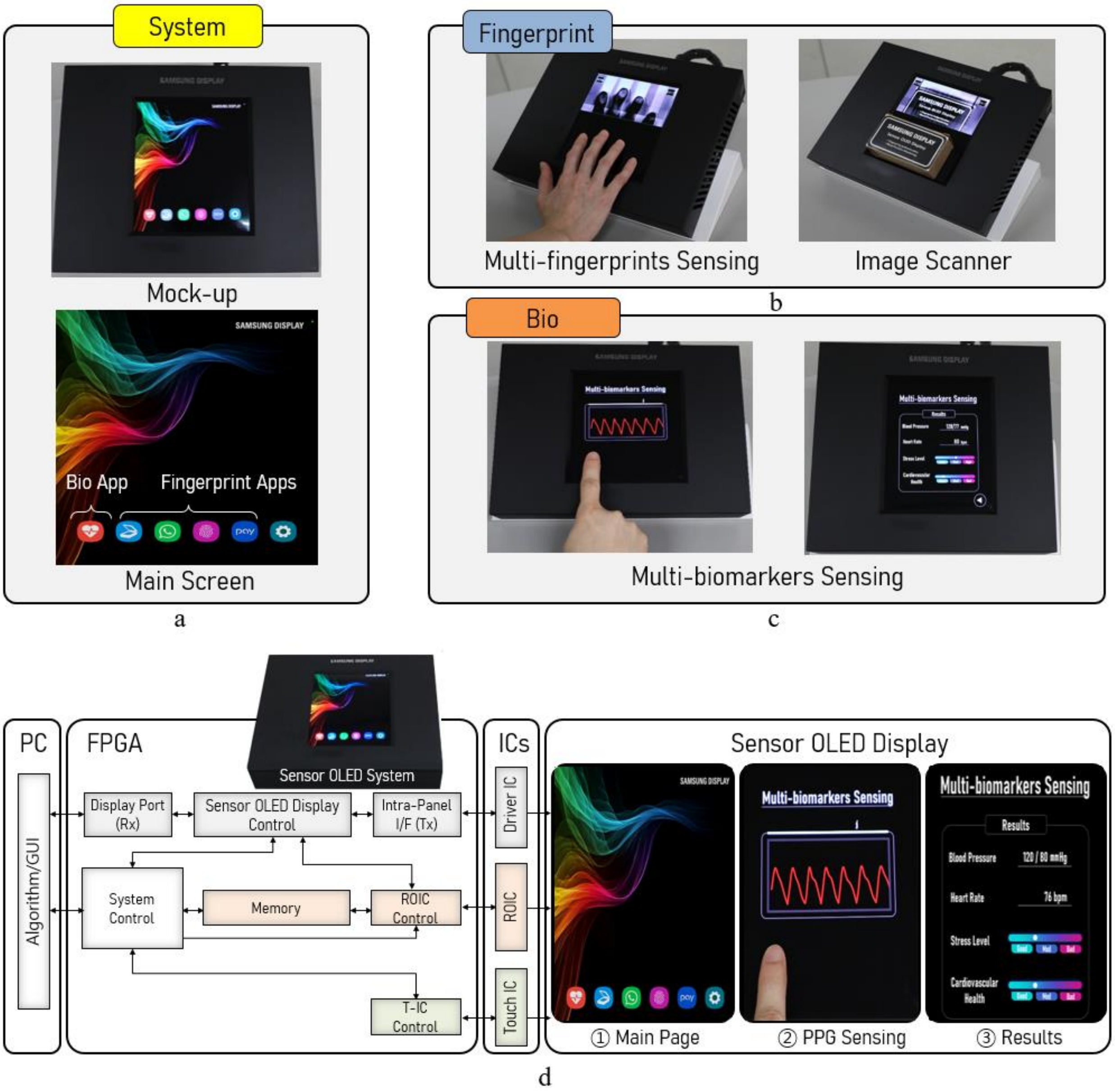

Supplementary Figure 6: The multiple objects sensing OLED. a, Implemented system with the Sensor OLED and main screen, **b~c,** Application examples of the multiple objects sensing (fingerprint such as a multiple-fingerprints sensing, image scanner and multiple-biomarkers including BP, HR, HRV, CH), **d**, Mock-up system architecture.

Sensor Signal Vs Image Signal

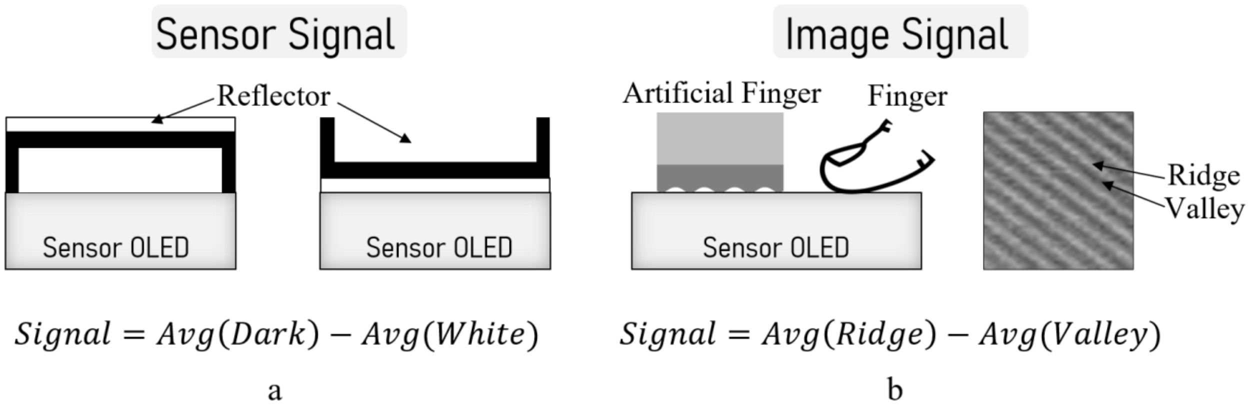

Supplementary Figure 7: Sensor signal Vs Image signal. a, Sensor signal is the code difference between black and white images obtained while the reflectors are on the Sensor OLED. It represents the full dynamic range of the OPD. **b,** Image signal is the code difference between ridge and valley images obtained while the artificial finger is placed on the Sensor OLED. It indicates the signal range of the OPD in the fingerprint application.

Concept of the Multi-fingers Authentication

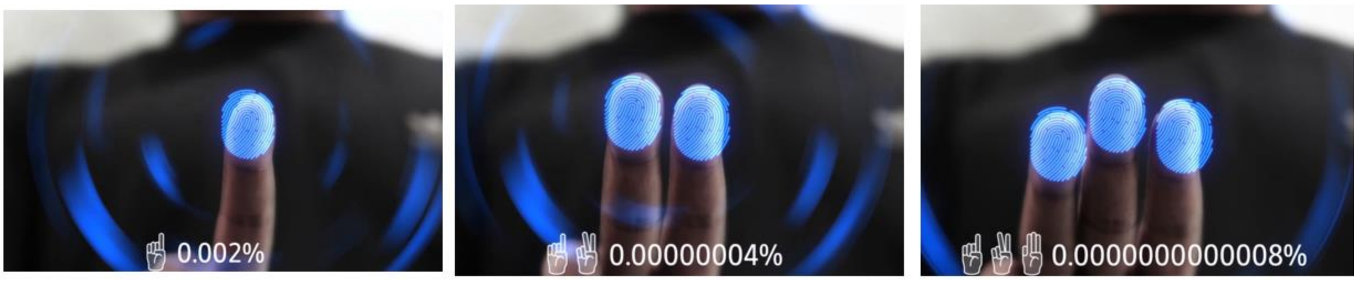

Supplementary Figure 8: The concept of the multi-fingers authentication It can abruptly improve a security level and the approximately estimated FARs are shown in the figure depending on the fingers used for the authentication.

Bio sensing applications

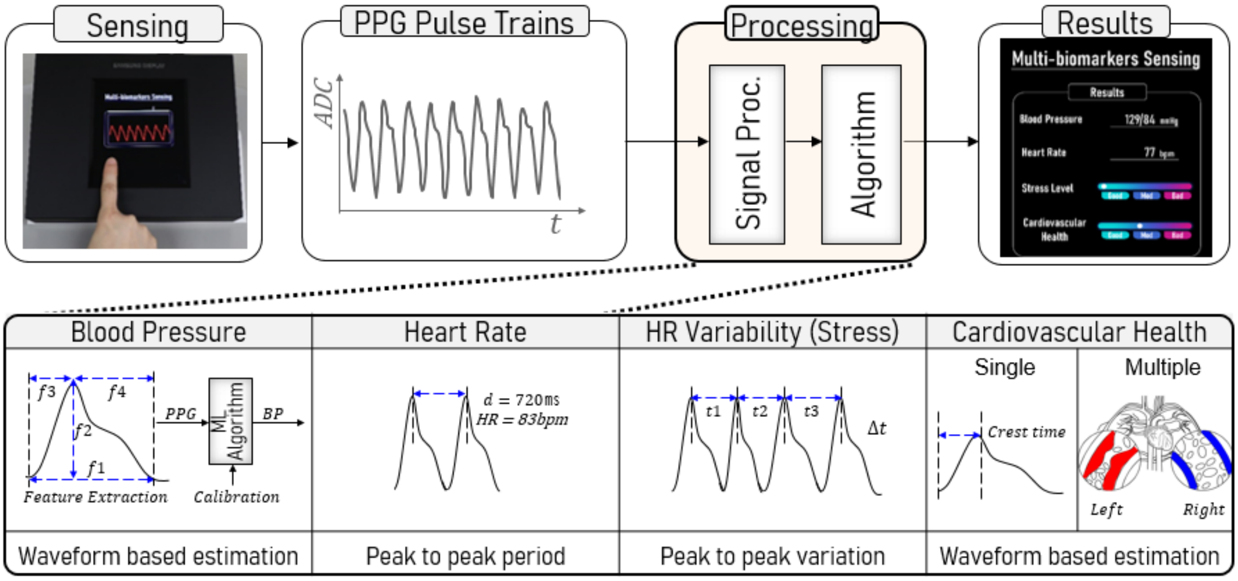

Supplementary Figure 9: The multiple biomarkers sensing applications. The principle of the multiple-biomarkers sensing is shown. Based on the PPG pulse trains obtained, the distinct pulse features, period, peak-to-peak variations, low frequency information etc. are extracted to estimate the relevant biomarkers

Pilot trial with the Sensor OLED.

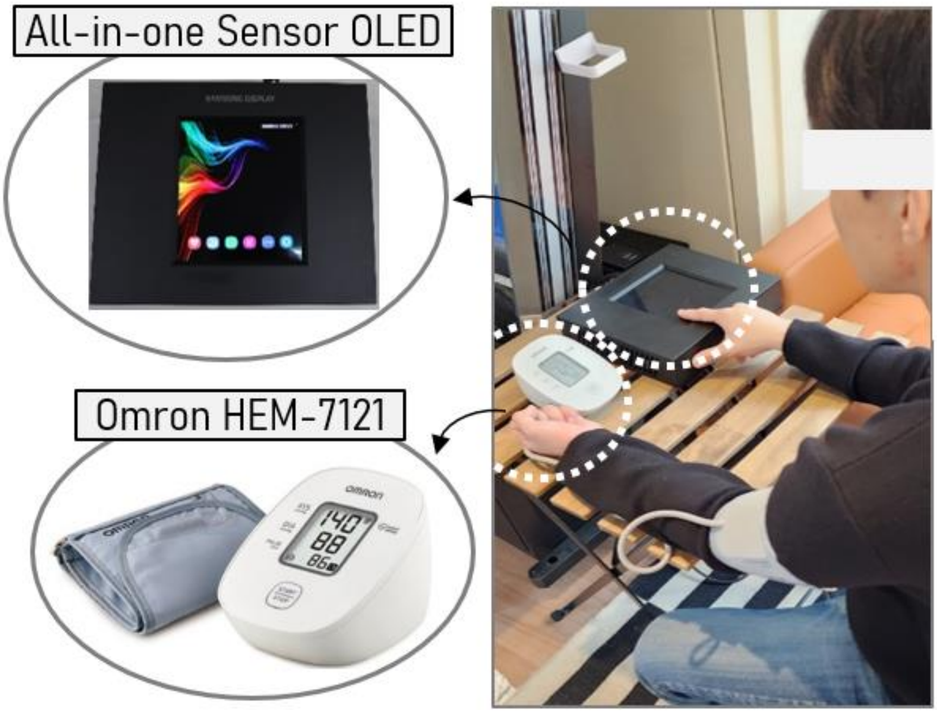

Supplementary Figure 10: The pilot trial test environment. Concurrent measurements were executed from the reference device (Omron HEM-7121) and the implemented system with the Sensor OLED from the each participant as shown in the figure. The data from the 32 participated users were gathered and used for the algorithm and accuracy test.

Blood Pressure Monitoring Technologies

	Method	Principle	Pros	Cons	
Require Calibration	Pulse Transit Time [1]	Time delay between proximal (ECG) and distal (PPG) arterial waveforms	Solid theory, Continuous BP monitoring	Require periodic calibration	Sensors at the two measurement sites
	Pulse Wave Analysis [2]	Extract Blood pressure related features from an arterial waveform and estimate BP with the machine learning	Use of single PPG sensor, Continuous BP monitoring, Approved by the regulatory		Lack of theory
	Facial Video Processing [3]	Extract pulse waveform from the facial video stream obtained by the cameras	Easy access (PPG: Camera from Smartphone, BP Estimation: App in Smartphone)		Low waveform quality
Not require Calibration	Oscillometric Finger Pressing [4]	Press the fingertip against a sensor unit which is embedded in the smartphone	Easy access (Sensor: Smartphone BP Estimation: App in Smartphone)	Require user action (precise pressure control)	
	Volume Control [5]	Adopting cuff-based volume clamping principle	Continuous BP monitoring	Require cuff and actuator (Disturbing, Finger numbness)	

*ECG: Electrocardiogram

Supplementary Table 1: Blood pressure monitoring technologies [6, 7] This table summarized the BP monitoring technologies. It categorized depending on the requirement of user calibration. The sensing principle and its characteristics are briefly tabulated. In this paper, we approached the BP estimation algorithm based on the PWA, as it is more suitable for interfacing with the Sensor OLED. The BP related features are extracted from the obtained PPG and the BP is estimated from the machine learning based algorithm.

Supplementary References

1. Mukkamala, R. et al. Toward Ubiquitous Blood Pressure Monitoring via Pulse Transit Time: Theory and Practice. *IEEE Transactions on Biomedical Engineering,* **62** 1879-1901(2015)

2. MF, O'Rourke. et al. Pulse wave analysis. *Journal of hypertension. Supplement: Official Journal of the International Society of Hypertension,* **14(5)** S147-57(1996)

3. Schoettker, P. et al.Blood pressure measurements with the OptiBP smartphone app validated against reference auscultatory measurements.*Sci Rep* **10**, 17827(2020)

4. Chandrasekhar, A. et al.An iPhone Application for Blood Pressure Monitoring via the Oscillometric Finger Pressing Method.*Sci Rep* **8**, 13136(2018)

5. Fortin, J et al: Continuous non-invasive blood pressure monitoring using concentrically interlocking control loops. *Computers in Biology and Medicine.* **36,** 941–957 (2006)

6. Mukkamala, R.et al. Cuffless Blood Pressure Measurement, *Annual Review of Biomedical Engineering.* **24,** 203-230 (2022)

7. Solà, J. et al. The Handbook of Cuffless Blood Pressure Monitoring. Springer (2019).
