# Supplementary material for: Sensor organic light-emitting diode display, combining fingerprint and biomarker capturing
Source: Commun Eng. 2024 Jul 4;3:92. doi: 10.1038/s44172-024-00239-8 (PMC11224248; doi:10.1038/s44172-024-00239-8)
Supplement: Supplementary file 2 — Description of Additional Supplementary Files [file 44172_2024_239_MOESM2_ESM.pdf]

Description of Additional Supplementary Files

File name: Supplementary Movie 1.

File description: Video demonstration of the Multi-functional Sensor OLED operation.
